# Mapping the architecture of the temporal artery with photoacoustic imaging for diagnosing giant cell arteritis

**DOI:** 10.1016/j.pacs.2022.100384

**Published:** 2022-07-04

**Authors:** Magdalena Naumovska, Aboma Merdasa, Björn Hammar, John Albinsson, Ulf Dahlstrand, Magnus Cinthio, Rafi Sheikh, Malin Malmsjö

**Affiliations:** a Department of Clinical Sciences Lund, Ophthalmology, Lund University, Skåne University Hospital, Lund, Sweden; b Department of Biomedical Engineering, Faculty of Engineering, Lund University, Lund, Sweden

**Keywords:** Photoacoustic imaging, Giant cell arteritis, Unsupervised spectral analysis, Noninvasive, clinical diagnosis

## Abstract

Photoacoustic (PA) imaging is rapidly emerging as a promising clinical diagnostic tool. One of the main applications of PA imaging is to image vascular networks in humans. This relies on the signal obtained from oxygenated and deoxygenated hemoglobin, which limits imaging of the vessel wall itself. Giant cell arteritis (GCA) is a treatable, but potentially sight- and life-threatening disease, in which the artery wall is infiltrated by leukocytes. Early intervention can prevent complications making prompt diagnosis of importance. Temporal artery biopsy is the gold standard for diagnosing GCA. We present an approach to imaging the temporal artery using multispectral PA imaging. Employing minimally supervised spectral analysis, we produce histology-like images where the artery wall is clearly discernible from the lumen and further differentiate between PA spectra from biopsies diagnosed as GCA- and GCA+ in 77 patients.

## 1 Introduction

Giant cell arteritis (GCA) is the most common form of primary vasculitis in the elderly, presenting with symptoms including headache, jaw claudication, fever and fatigue [1], [2], [3], [4]. If left untreated, GCA can result in significant morbidity, including blindness, or death. Early diagnosis and treatment are therefore of utmost importance [2], [3]. The diagnosis of GCA is primarily based on clinical evaluation, and is confirmed by temporal artery biopsy, an invasive procedure where a segment of the temporal artery is surgically excised and examined histopathologically to identify inflammatory lesions in the vessel wall. Temporal artery biopsy is associated with several risks, such as injury to the facial nerve or the trigeminal nerve, hemorrhage, wound infection and scarring [5], [6], [7]. It would, therefore, be of great advantage to develop a non-invasive method capable of mapping the extracranial vascular network, as well as other blood vessels, in order to improve the diagnostic accuracy, while at the same time avoiding surgery and treatment with potentially harmful side-effects.

Non-invasive methods of diagnosing GCA have been investigated previously, although each has limitations. Color Doppler ultrasonography has insufficient diagnostic accuracy and correct diagnosis depends on the expertise of the operator [8], [9], [10]. Magnetic resonance imaging and positron emission tomography with flourodeoxyglucose have also been investigated, but have both been found to have insufficient specificity and sensitivity in diagnosing GCA [11]. These vascular imaging modalities require the use of a contrast agent, or exposure to ionizing radiation, have poor usability or low resolution, or a combination of both [12]. Although these techniques can support the diagnosis of GCA, they cannot replace surgical biopsy and histopathological examination [5], [11], [13], [14]. Hence, there is to date no reliable non-invasive method of diagnosing GCA.

Photoacoustic (PA) imaging is a rapidly developing biomedical imaging technique, that combines the strengths of optical and ultrasound imaging to reveal the molecular composition of tissue at high resolution [15], [16]. In PA imaging, energy from non-ionizing laser pulses is absorbed by endogenous chromophores, causing a thermoelastic expansion that generates acoustic waves, which are detected by an ultrasound transducer [17], [18]. PA imaging can provide high-resolution three-dimensional (3D) images of the structure and function of tissues, including small blood vessels in animals [19], where in humans some examples involve vessels of the skin [20], [21], coronary arteries [22], the radial artery [23], [24], the tibialis posterior and dorsalis pedis arteries [25], the carotid artery [26], [27], the digital arteries [28], [29], and the palmar digital arteries [30], [31]. In most of these studies, imaging of the vascular network was based on detection of oxygenated and deoxygenated hemoglobin, which strongly absorb light of different wavelengths, thus providing good PA contrast. GCA is an inflammatory disease involving the infiltration of leukocytes into the vessel wall, which leads to a reduced lumen and thus restricted blood flow. The detection of hemoglobin inside the artery is therefore not sufficient for diagnosing GCA, and new methods of characterizing the artery wall are needed.

The aim of the present study was to use multispectral PA imaging for detailed spectral and spatial characterization of the temporal artery *ex vivo*. Temporal artery biopsies were investigated directly after surgical excision from 77 patients suspected of having GCA. Spectral analysis was used to generate histology-like images from the multispectral PA images of the temporal artery cross-sections, providing detailed spectral and spatial information on the architecture of the artery wall.

## 2 Materials and methods

### 2.1 Subjects

Patients with suspected GCA undergoing temporal artery biopsy at the Department of Ophthalmology at Skåne University Hospital, in Lund, Sweden, were included in the study from October 2017 to December 2020. PA imaging was performed *ex vivo* on the temporal artery specimens before the histopathological examination. Some of the specimen were also examined with high-frequency ultrasound center frequency shift (CFS), as described in our previous study [32].

Subjects were excluded if the biopsy was inconclusive (i.e. uncertainty of the presence of inflammatory changes in the vessel wall or inappropriate biopsy specimen) or if the signal-to-noise ratio in the PA images was insufficient for reliable analysis. Ninety patients were assessed for eligibility and of these 13 were excluded due to the exclusion criteria. Out of these, ten biopsies were excluded due to limited SNR. The reason for the low SNR was that we collected patient data for 3 years and in the beginning, some acquisition parameters were not optimized which yielded a poor SNR and the biopsies were therefore excluded. One subject declined participation. A total of 77 subjects were thus included in this study. Patient characteristics are given in Table 1.

**Table 1:** Patient characteristics.

	All patients (n = 77)	GCA+ biopsies (n = 36)	GCA- biopsies (n = 41)
Gender (female/male)	57/20	25/11	32/9
Median age (range) in years	73 (52–90)	75 (61–90)	72 (52–83)
GCA treatment with corticosteroids prior to biopsy (n)	68	32	36
Eye complications due to GCA (n)	10	10	0
Systemic hypertension (n)	37	13	24
Diabetes mellitus, type 1 or type 2 (n)	14	6	8
Previous cardiovascular events (n)	6	0	6
Previous cerebrovascular events (n)	8	5	3
Diagnosis of polymyalgia rheumatica prior to temporal artery biopsy (n)	10	3	7
Current or previous smokers (n)	40	18	22

There was some degree of variability in spectral characteristics between the different temporal artery biopsies. However, the limited study size made subgroup analysis impossible. It would be of great interest to assess the effect of medical conditions and treatments that may have affected the vasculature, such as diabetes, cardio- and cerebrovascular disease or smoking, or the duration of cortisone treatment, on the spectral signature, in the future.

### 2.2 Photoacoustic image acquisition

**Fig. 1:**
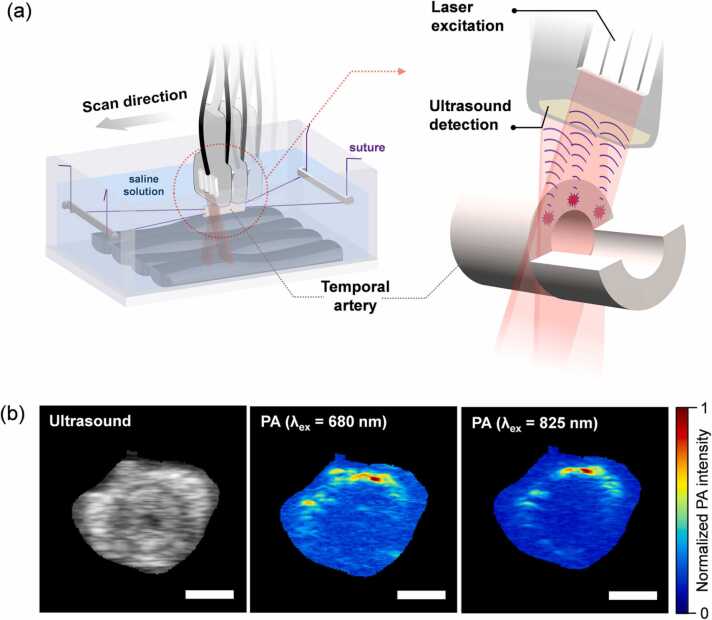
(a) Illustration of the photoacoustic (PA) scanning setup used to examine the temporal artery biopsies. The artery is mounted in a Perspex container using surgical sutures and the container is filled with balanced salt solution. The artery is then scanned with the PA probe, consisting of an ultrasound transducer with laser fiberoptic bundles attached on each side, which is fixed to an adjustable arm with a linear stepper motor to scan the whole specimen. The biopsy is irradiated with pulsed laser light, causing thermoelastic expansion, which generates acoustic waves that can be detected by the ultrasound transducer. (b) Representative examples of a cross-sectional ultrasound image of the temporal artery (left), and the corresponding PA images acquired at excitation wavelengths of 680 nm (middle) and 825 nm (right). The differences in the PA intensity distribution between the two PA images indicate a difference in tissue composition. Scale bar = 1 mm.

Temporal artery biopsy was performed at the Department of Ophthalmology in Lund, Sweden, under local anesthesia according to local practice. The biopsy was then placed in balanced saline solution (BSS). The vessel lumen was first rinsed with BSS, using a hypodermic needle and a syringe, to remove blood from the vessel. Sutures were sewn to each end of the biopsy, and it was then placed in a 100 × 70 × 50 mm Perspex container filled with balanced salt solution (BSS), with a layer of black ultrasound-attenuating material in the bottom. The biopsy was held in place by the sutures. The setup and scanning of the temporal arteries are illustrated in Fig. 1a.

PA imaging was performed using a Vevo LAZR-X imaging system (FUJIFILM VisualSonics Inc., Toronto, ON, Canada) equipped with an ultrasound linear array transducer and a tunable laser with nanosecond pulse duration. The ultrasound transducer (MX400) operates at a central frequency of 30 MHz with a bandwidth of 22–46 MHz, providing axial and lateral resolutions of 50 µm and 110 µm, respectively. The pulsed laser operates at 20 Hz, and the pulse duration is on the order of a few ns. The laser is spectrally tuned between 680 and 970 nm in steps of 5 nm to excite the sample with 59 unique wavelengths, which generates 59 unique PA images, or one multispectral PA image.

Two planar light beams, located on either side of the ultrasound linear array, illuminate the temporal artery biopsy (see Fig. 1a). A 10 mm thick Aquaflex Ultrasound gel pad (Parker Laboratories Inc., Fairfield, CT, USA) with protective plastic film was used to achieve an adequate distance between the laser fibers and the temporal artery, as described previously [33]. The transducer was fixed to an adjustable arm (Mounting Accessory, GCX Corporation, Petaluma, CA, USA) and the holder was driven by a linear stepper motor (VisualSonics Inc., Toronto, ON, Canada) with a step size of 0.5 mm, to allow multispectral PA images to be captured over a larger volume, resulting in a 3D stack of data [34].

A multispectral PA image contains a spectrum with 59 spectral elements in each pixel of the image. The length of the biopsy determined how many cross-sections we could measure, where on average we acquired a little more than three measurements per biopsy. In the collective analysis, we included all measurements from all biopsies, which is why we got a total 259 data points even though the number of patients was 77. When linear scanning was performed to image a volume, the number of spectral components was reduced to twelve in order to reduce the measurement time and the amount of data. Multiple excitation wavelengths provide detailed absorption spectra of different tissue chromophores, allowing a more robust analysis when determining the distribution of chromophores, as well as the identification of multiple chromophores with distinct spectral features. However, care must be exercised when reducing the amount of spectral information to avoid missing any unique spectral features in the data. Therefore, we reduced the spectral resolution while essentially maintaining the spectral range (680–940 nm in steps of 20 nm, excluding 920 nm). Fig. 1b shows the ultrasound image of the artery and the spectral absorption at different wavelengths in the PA images.

### 2.3 Data import and pre-processing

**Fig. 2:**
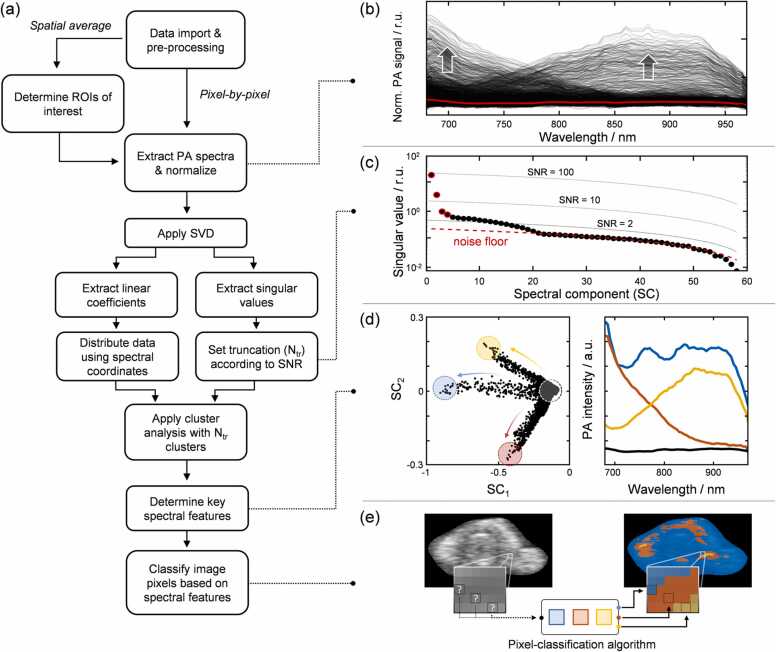
(a) Flow chart providing an overview of each step in the analysis. Initially, spectra are extracted on either a pixel-by-pixel basis or as an average of several regions of interests (ROIs), which results in a number of spectra to be compared. (b-c) SVD is then applied to determine the number of spectral features present in the data set, using the relative strength of the singular values to determine where a cut-off (truncation) should be set. Each spectrum can be placed in a new coordinate system based on its spectral features as single point (d, left), where the distance between two points reflects spectral (dis)similarity. The four spectra to the right are averaged from the spectra extracted from the spectral coordinates at the ends of the clusters indicated with colored circles, as well as the region connecting them (black). (e) Finally, having determined the key spectral features from the spectral clustering, a pixel-classification algorithm is used to color code each pixel based on which spectral feature it most resembles in order to create a new image that results in the different spectral features seen in the PA image.

A flow chart describing the analysis process is shown in Fig. 2a, and examples of the results obtained in Fig. 2b-e. Raw data were exported from VisualSonics Vevo LAB 3.1.0 software and imported into MATLAB v.2017b (MathWorks Inc., Natick, MA, USA) where the analysis was performed. The signal from the artery was obtained by removing the background from the images as described previously [35]. The data were then prepared for spectral analysis, which involved extracting spectra on a pixel-by-pixel basis, or spatial averaging in order to obtain a spectrum from either an entire cross-section of the temporal artery or a smaller region within that cross-section. The spectra were normalized by dividing by the mean PA intensity of the entire image. This allowed spectra from one measurement to be compared to spectra from other measurements with less dependence on acquisition parameters such as laser power and signal gain.

### 2.4 Singular value decomposition

Fig. 2b shows an example of 500 randomly extracted PA spectra from individual pixels in a cross-sectional PA image of an artery similar to Fig. 1b. The red line represents the average PA spectrum, which clearly does not capture the two distinct spectral features at both shorter and longer wavelengths. To determine the number of spectral features contained in the spectral data set, singular value decomposition (SVD) was used to decompose the entire data set (M) into a set of eigenvectors (V), singular values (∑), and linear coefficients (U) according to $M=U\Sigma V^{*}$, where the asterisk indicates the transpose.The purpose of this is to construct a new coordinate system that better represents the spectral variance contained in the data set. In other words, the data set is sorted such that important spectral features become isolated making them more visible. These spectral features are represented by the basis vectors (or spectral components, SCs) that are ranked from the most to the least important. This means that all the important information in the data set is contained in the first few SCs, while the later ones do not contribute any useful information, and can be regarded as data noise.

The singular values provide a measure of the relative importance of each SC, and are used to determine how many important spectral features there are in the data set. Fig. 2c shows the singular values plotted for each spectral component in black dots. Using the last spectral components (excluding the last few points), which represent the noise in the data, the red dashed line is extrapolated toward the first spectral components in order to determine the “noise floor”. This serves as a reference point when determining the signal-to-noise ratio (SNR) of the singular value of each spectral component. The SNR can thereafter be used to determine how many spectral components are relevant in the description of the main spectral features of the data set (truncation). In the example data set, an SNR = 3 truncates the data set into four relevant spectral components, which are highlighted with red circles in Fig. 2c. For the sake of consistency, we use SNR = 3 in situations where SVD analysis is employed on spectra acquired as an average over one cross-section, and SNR = 2 when a single cross-section is examined pixel-by-pixel. The latter selection is to increase spectral variability.

### 2.5 Spectral clustering

Each measured spectrum in M has a linear coefficient for each of the spectral components, which in this example has been reduced to four. Thus, the spectral variance of all 500 spectra should be found within these four vectors. While it is mathematically possible to construct a 4D orthonormal coordinate system, it is challenging to represent this visually. Therefore, the presentation is limited to two dimensions represented by the linear coefficients for the first spectral component (SC_1_) on the x-axis, and the linear coefficient for the second spectral component (SC_2_) on the y-axis. Each of the 500 PA spectra is represented by a black dot in Fig. 2d (left). In this plot, the coordinates reveal how much of the two main spectral features each spectrum contains. It can be seen in this plot that the data are grouped in three directions, indicating that the data contain three main spectral features.

The highest density of data points is found closest to the origin of the coordinate system. Taking the average of all the PA spectra associated with the data points within the ROI close to the origin, results in a spectrum similar to the average PA spectrum for the entire data set in Fig. (2b). Repeating this process for the other three ROIs, shaded yellow, blue, and red in Fig. 2d, gives three distinct spectra, two of which (the red and yellow) show similar features to the trends observed in Fig. 2b. The third spectrum (blue) appears to be a combination of the two, which makes sense from the perspective of the new coordinate system since this corresponds to the distribution of data points in the middle of the upper and lower clusters. In other words, this cluster (blue) primarily shows variance along SC_1_, while the other two exhibit variance in both SC_1_ and SC_2_. SVD thus allows a more suitable coordinate system to be created in which different spectral features become more visible.

### 2.6 Spectral mapping

Instead of manually selecting ROIs as demonstrated in Fig. 2d, we employed k-means clustering, which divides the data points into a set number of clusters based on the relative distances between all the points. Since distance in the new coordinate system reflects spectral (dis)similarity, this allows for the implementation of a classification algorithm that assigns a class to each data point based on spectral features, and thereafter codes the corresponding pixel from which the spectrum was extracted with a unique color (Fig. 2e). It is thus possible to generate new images that show differences in PA spectral features in different colors. Spectral mapping can realistically only be applied when using pixel-by-pixel spectral extraction, while the steps before pixel classification can be applied when spatial averaging has been carried out.

### 2.7 Histopathological examination

After PA scanning, the temporal artery biopsy specimens were placed in formalin and sent to the pathology laboratory. The biopsies were cut into 3 mm sections, and these were then sectioned into 3 parts, each with a distance of about 200 µm between them. Three micrometer thick sections were cut from each part and stained with hematoxylin-eosin and elastica van Gieson for histological examination.

### 2.8 Statistical analysis

Calculations and statistical analysis were performed using GraphPad Prism 9.2 (GraphPad Software Inc., San Diego, CA, USA). An Anderson-Darling test showed normal distribution of the data (p = N.S.). Thereafter the statistical difference between the spectra in the wavelength range from 680 nm to 970 nm, from the cluster containing GCA- and GCA+ samples, was analyzed using two-way ANOVA with Bonferroni’s multiple comparisons test used for comparisons between groups. Significance was defined as p < 0.05.

## 3 Results

### 3.1 Histopathology

**Fig. 3:**
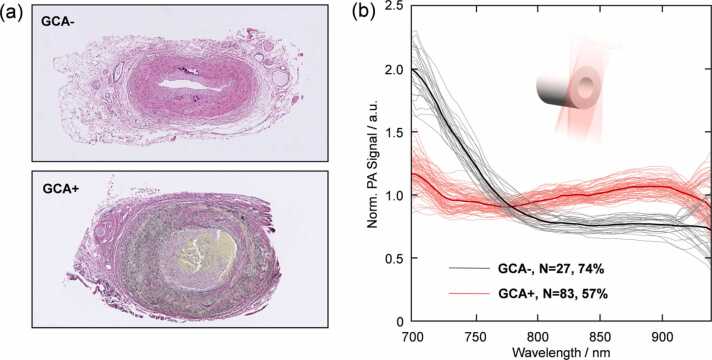
(a) Histopathological sections of a GCA- and a GCA+ temporal artery, stained with hematoxylin-eosin and elastica van Gieson staining. (b) PA spectra extracted as a spatial average from individual cross-sections of the artery (schematic inset). The light traces represent all the spectra from the cluster containing most GCA- (black) and GCA+ (red) samples, while the heavy traces represent the mean spectrum of each cluster. The spectra of the clustered groups showed a significant difference at all wavelengths between 680 and 970 nm (p < 0.0001) except in the range 770–785 nm, which is where they intersect, where it was not significant.

Of the 77 patients included in this study, 36 had biopsies with histopathological signs of GCA (GCA+), while 41 biopsies showed no signs of GCA (GCA-). Typical signs of GCA+ were infiltration of inflammatory cells (e.g. leukocytes), multinucleate giant cells, artery lumen reduction and fragmentation of the internal elastic lamina, visualized by elastica van Gieson staining (Fig. 3a).

### 3.2 The spectral signature

The spectral signature of temporal arteries was extracted as a spatial average over the cross-section of the artery (see schematic inset in Fig. 3b). Spectra from a total of 259 cross-sections were analyzed (a few per patient), each of which was tagged with their corresponding biopsy result, being either GCA- or GCA+ . The spectral analysis outlined above and in Fig. 2 yielded five different clusters based on distinct spectral features. The percentages of spectra belonging to a cluster that was histopathologically diagnosed as either GCA- or GCA+ were then determined. We found one cluster containing 27 spectra, of which 74% were GCA-, while another cluster contained 83 spectra, 57% of which were GCA+ . The spectra from the clusters containing most GCA- and GCA+ samples were significantly different (p < 0.0001, Fig. 3b).

### 3.3 Spatially resolved spectra

**Fig. 4:**
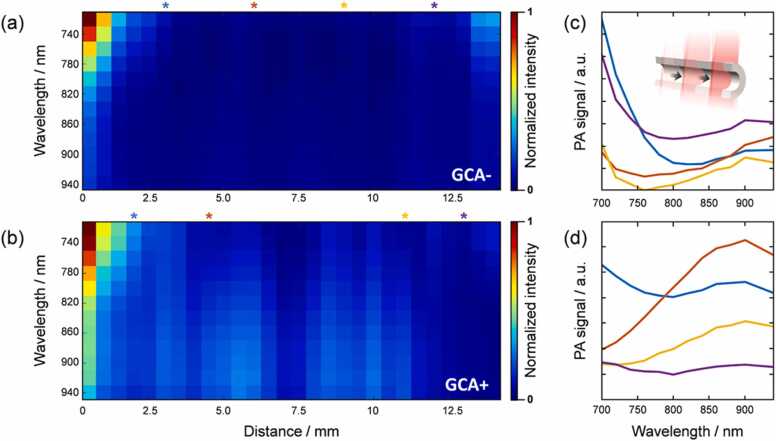
Spatio-spectral heat map showing the spectral variation along the length of a GCA- artery (a) and a GCA+ artery (b). The distance along the length of the artery is given on the x-axis, and the color-coded normalized PA intensity on the y-axis. Individual spectra were extracted from four different locations along the length of the GCA- (c) and the GCA+ (d) temporal artery (schematic inset), showing the variability in the spectra along the length of the artery.

To obtain a more complete spectral picture of a single artery, multiple PA image stacks were acquired sequentially from different locations along the temporal artery (see schematic inset in Fig. 4c). A single PA spectrum was generated at each location, after which they were stitched together to generate a spatio-spectral heat map (Fig. 4a and b). This provides an image in which the spectral variation along the length of the artery is better visualized, allowing the spectral differences between GCA- and GCA+ arteries to be seen more clearly. There was significant variability in the spectra extracted from different locations along the long axis of the artery, which could potentially reflect the histopathological appearance of so-called skip lesions [36], [37].

### 3.4 Pixel-by-pixel analysis in a cross-section of the artery

**Fig. 5:**
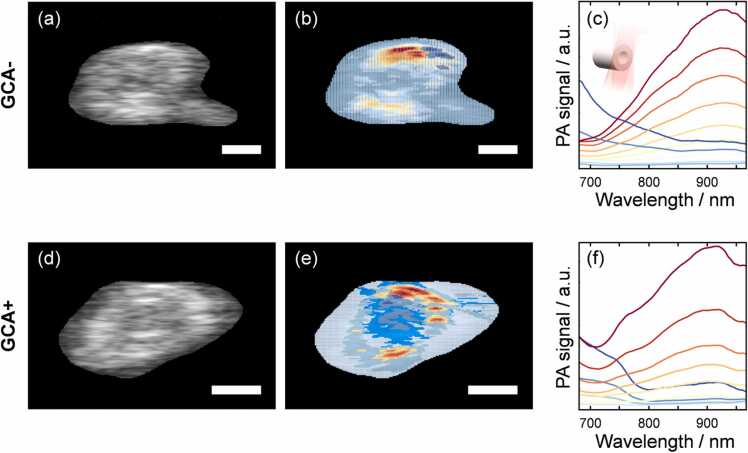
Cross-section of a GCA- sample showing (a) the ultrasound image, (b) color coded image generated from the pixel-classification algorithm in which each color represents a spectral feature that is shown in (c). (d-f) show the same results for a GCA+ sample. The schematic inset in panel (c) illustrates the measurement geometry. The scale bars represent 1 mm.

To further explore the extent to which the PA spectra may vary spatially, we performed pixel-by-pixel classification analysis of the cross-sectional PA images. Fig. 5 shows representative examples for GCA- and GCA+ arteries, where each pixel has been color-coded based on distinct spectral features. In both examples, the spectral features vary radially from the center, which is expected from an anatomical perspective considering the structures of the artery, such as the lumen, the vessel wall and the adventitia. In the GCA- sample, the center region is primarily represented by PA spectra of low intensity. This is in contrast to the GCA+ sample, where the central region of the artery exhibits a stronger signal and yields a PA spectrum that is visibly different from the corresponding region of the negative sample. These observations agree well with the histological images in Fig. 3a.

### 3.5 Pixel-by-pixel analysis in a longitudinal section of the artery

**Fig. 6:**
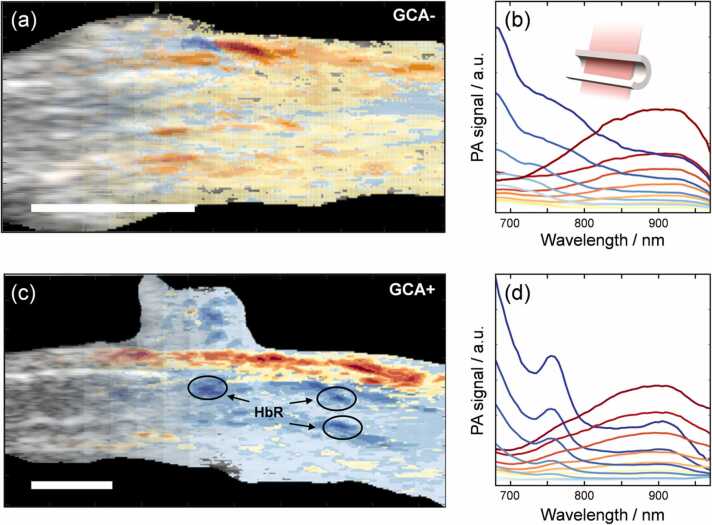
(a) Longitudinal section of a GCA- sample after the pixel-by-pixel classification based on spectral features, which are shown in (b). The color-coding in (a) and (b) are the same. (c-d) corresponding results for a GCA+ sample. The schematic inset in panel (c) illustrates the measurement geometry. The scale bars represent 2 mm. The three highlighted regions in the GCA+ sample where there is a strong PA spectral signature of deoxygenated Hb (HbR) are likely remnants of blood that was not rinsed out prior to investigation.

Spectral variation is also expected along the entire artery, and a single cross-sectional measurement may not capture all the relevant information. PA imaging enables analysis of the spectral information on a pixel-by-pixel basis in a longitudinal section of the artery. This provides a spatially resolved map of the architecture of the vessel showing the anatomical structures of the artery and the respective spectral features. Fig. 6 shows such examples for a GCA- and a GCA+ artery, together with the spectral features of the different regions of the sample.

## 4 Discussion

We have shown that photoacoustic imaging, in combination with advanced numerical analysis, can be used to obtain detailed spatially resolved PA spectra from temporal arteries, providing important information on the artery wall. The images produced by the spectral analysis and pixel-classification algorithm show strong resemblance to those observed histopathologically. This is promising for the clinical implementation of PA imaging as it enables visualization of the artery wall architecture. Although the spatial resolution of PA imaging cannot yet compete with that of histopathological examination, the spectral information provided by PA imaging reveals the molecular composition of tissue, surpassing that which can be obtained with histology.

We used 59 excitation wavelengths when acquiring and analyzing the multispectral PA images. Most previous PA imaging studies on human blood vessels [20], [21], [23], [25] have used a limited number of excitation wavelengths in the analysis which may be sufficient, for example, to assess oxygen saturation, but is only useful if the expected spectral variance is known prior to the measurements. In other words, if the chromophores that may be present in the sample are known, spectral unmixing at a few wavelengths can be utilized to determine the relative abundance of those chromophores. In cases where the spectral variation is unknown, the maximum number of available excitation wavelengths should be used in the spectral analysis. The only previously reported study on the artery wall, using a larger number of excitation wavelengths (25 in the spectral range between 1130–1250 nm), was performed on the carotid artery after excision from cadavers, which enabled the identification of fat and collagen, which are typical indicators of plaque [22]. The present study is a first study of its kind, mapping the temporal artery with full spectral range, without having any previous guidance of what spectral changes to expect in GCA- and GCA+ arteries.

The presented spectral analysis employed in this study revealed an interesting aspect besides the interesting spectral features of the temporal artery with high spatial resolution. In the analysis outlined in Fig. 2a, pixel-by-pixel analysis was first used to extract the PA spectra, after which spectral analysis was applied and finally the pixels were color-coded based on the spectral features. It should be noted that the spectra were analyzed without considering their spatial coordinates in the PA image. It is only after the spectral analysis that the pixels are color-coded, and it is therefore intriguing that we not only observe gradual spectral changes that correlate with neighboring regions in the image, but also that these reveal patterns that coincide with anatomical structures. The only instruction provided in the spectral analysis is the SNR that should be used to determine how many important spectral features there are in the data set, and the same SNR is used for the analysis of all samples. In the pixel-by-pixel analysis, an SNR = 2 was used to generate more spectral variability and yielded an average of 5.37 + /- 1.88 spectral components across all 77 samples. Apart from selecting the SNR, the approach is completely blind, unlike similar studies on microvascularization using PA imaging, where spectral unmixing is often used [38].

We examined a total of 259 temporal artery cross-sections from 77 patients, which is a far greater number of subjects than in any previous studies. In order to differentiate GCA- from GCA+ biopsies by PA imaging, all the spectra needed to be clustered into five different groups, of which the spectra in the cluster with the most GCA- biopsies were significantly different from that with the most GCA+ biopsies. Although some spectral difference was seen between GCA- and GCA+ biopsies, the sensitivity must be improved. In its current state of development, the spectral mapping of the temporal artery cannot alone reliably diagnose GCA. Although the accuracy of the technique will probably be improved in the future, it is probable that PA imaging will have to be combined with clinical parameters obtained from patient examination to ensure diagnostic accuracy. Indeed, GCA is today mainly diagnosed based on temporal artery biopsy examination in combination with clinical parameters [39]. There are currently no specific diagnostic criteria or blood biomarkers for GCA [40], [41]. The American College of Rheumatology (ACR) criteria for the classification of GCA [42], and the European League Against Rheumatism (EULAR) recommendations for the management of large vessel vasculitis may assist in diagnosing GCA, but these are intended to distinguish patients with GCA from those with other forms of vasculitis, and cannot be used to diagnose GCA [43], [44]. The ACR and EULAR criteria require the fulfillment of 3 of 5 core features: age 50 years or older at onset, new onset of headache, clinical temporal artery abnormality, elevated erythrocyte sedimentation rate (ESR) of at least 50 mm/h and an abnormal temporal artery biopsy [42]. The importance of using clinical parameters is highlighted by the findings of the TABUL study (Temporal Artery Biopsy vs. Ultrasound in Diagnosis of GCA) [13]. One-third of the patients eventually diagnosed as having GCA had neither a positive ultrasound scan nor a positive temporal artery biopsy, emphasizing the importance of assessing clinical indicators to support the diagnosis.

The examination of temporal arteries *ex vivo* is a necessary step toward the ultimate goal of in vivo diagnostic examinations. However, a number of issues need to be addressed before the technique can be clinically implemented. The impact of absorbing chromophores in circulating blood and in tissue above the artery gives rise to “spectral coloring” and must be reduced. This is a phenomenon that occurs as photons travel deeper into tissue and undergo wavelength-dependent absorption [45]. Spectral coloring was not a problem in the current study, as we imaged the artery without any blood inside, or any tissue above. However, in in vivo measurements, melanin in the epidermis will strongly attenuate the fluence spectrum reaching the artery, which may affect the analysis. However, this mainly becomes an issue when performing supervised analysis methods, such as linear spectral unmixing, since these methods depend on there being a known spectral signature in the measured data. Spectral coloring produces increasingly more distorted spectra deeper into the tissue, which limits spectral unmixing. The minimally supervised approach to spectral analysis in this study, where the procedure first identifies the relevant spectral features present in the data, and thereafter differentiates them in space, is more resilient to spectral coloring since it focuses on finding any difference, rather than a specific difference. In the present study, the temporal artery was not imaged in vivo because of the unresolved technical challenges of spectral coloring when imaging blood vessels that have an intraluminal signal, from hemoglobin. This generates spectral absorption that is presumably orders of magnitude higher than that from the actual vessel wall. Even though algorithms accounting for spectral coloring with the potential to improve the analysis have been developed [46], [47], these are not sufficient to enable discrimination of spectral signal of the healthy artery, from that originating form biological inflammatory processes and leukocyte infiltration due to giant cell arteritis.

## 5 Conclusions

We have described how multispectral PA imaging can be utilized to map the architecture of the temporal artery wall providing detailed spectral information. A minimally supervised spectral analysis approach was described and shown to be useful in identifying and using unique spectral signatures in a sample without a priori knowledge of which chromophores may be present. Although the present results are encouraging, further technical development, including motion tracking and techniques to correct for spectral coloring, are needed to enable in vivo examination. A large clinical trial will then be required before PA imaging can be considered useful as a clinical diagnostic tool for GCA.

## Funding information

This study was financed by Skåne County Council Research Grants, the Swedish Government Grant for Clinical Research (ALF), 10.13039/501100003252Lund University Grant for Research Infrastructure, Crown Princess Margaret’s Foundation (KMA), 10.13039/501100011077Skåne University Hospital (SUS) Research Grants, the 10.13039/100012538Swedish Cancer Foundation, Friends of the Visually Impaired Association in the county of Gävleborg, a project grant from the 10.13039/501100007687Swedish Society of Medicine (SLS), the Foundation for the Visually Impaired in the County of Malmöhus, Lund Laser Center Research Grant, Carmen & Bertil Regnérs Foundation, IngaBritt and Arne Lundberg’s Research Foundation and the Swedish Eye Foundation, Cronqvist Foundation, 10.13039/501100007308Swedish Medical Association and Lund University grant for Research Infrastructure.

## Declaration of Competing Interest

The authors declare that they have no known competing financial interests or personal relationships that could have appeared to influence the work reported in this paper.
